# A survey of food bank operations in five Canadian cities

**DOI:** 10.1186/1471-2458-14-1234

**Published:** 2014-11-28

**Authors:** Valerie Tarasuk, Naomi Dachner, Anne-Marie Hamelin, Aleck Ostry, Patricia Williams, Elietha Bosckei, Blake Poland, Kim Raine

**Affiliations:** Department of Nutritional Sciences, Faculty of Medicine, University of Toronto, Toronto, ON M5S 3E2 Canada; Department of Epidemiology, Biostatistics and Occupational Health, McGill University, Québec City, Canada; Department of Geography, University of Victoria, Victoria, Canada; Department of Applied Human Nutrition, Mount Saint Vincent University, Halifax, Canada; Dalla Lana School of Public Health, University of Toronto, Toronto, Canada; School of Public Health, University of Alberta, Edmonton, Canada

**Keywords:** Food banks, Food insecurity, Canada

## Abstract

**Background:**

Food banks have emerged in response to growing food insecurity among low-income groups in many affluent nations, but their ability to manage this problem is questionable. In Canada, in the absence of public programs and policy interventions, food banks are the only source of immediate assistance for households struggling to meet food needs, but there are many indications that this response is insufficient. The purpose of this study was to examine the factors that facilitate and limit food bank operations in five Canadian cities and appraise the potential of these initiatives to meet food needs.

**Methods:**

An inventory of charitable food provisioning in Halifax, Quebec City, Toronto, Edmonton, and Victoria, Canada was conducted in 2010. Of the 517 agencies that participated in a telephone survey of their operations, 340 were running grocery programs. Multivariate regression analyses were conducted to determine the association between program characteristics, volume of service, and indicators of strain in food banks’ abilities to consistently achieve the standards of assistance they had established.

**Results:**

Extensive, well-established food bank activities were charted in each city, with the numbers of people assisted ranging from 7,111 in Halifax to 90,141 in Toronto per month. Seventy-two percent of agencies indicated that clients needed more food than they provided. The number of people served by any one agency in the course of a month was positively associated with the proportion of food distributed that came from donations (beta 0.0143, SE 0.0024, p 0.0041) and the number of volunteers working in the agency (beta 0.0630, SE 0.0159, p 0.0167). Food banks only achieved equilibrium between supply and demand when they contained demand through restrictions on client access. When access to assistance was less restricted, the odds of food banks running out of food and invoking measures to ration remaining supplies and restrict access rose significantly.

**Conclusions:**

Despite their extensive history, food banks in Canada remain dependent on donations and volunteers, with available resources quickly exhausted in the face of agencies’ efforts to more fully meet clients’ needs. Food banks have limited capacity to respond to the needs of those who seek assistance.

## Background

Food scarcity and food deprivation have come to be recognized as a growing problem among low-income groups in many affluent nations [1–8]. Variously termed ‘food poverty’ or ‘household food insecurity’, this problem is now routinely monitored in some countries, and there is an abundance of research documenting its deleterious effects on health (e.g., [6, 9–16]). Household food insecurity is inextricably linked to socio-economic disadvantage [3, 5, 17–22], and it has been often attributed to social policy reforms that have diminished publicly funded supports for low-income households [2, 23, 24]. Recent research suggests that rising food and fuel costs are also contributing factors [25–28]. In concert with changing economic conditions and social policy reforms, charitable food assistance programs have emerged as an integral part of the response to food insecurity in many affluent countries, often organized around the concept of a ‘food bank’ [2, 3, 7, 24, 29–40]. While the operating structures of food banks differ across countries, particularly with respect to the extent of public sector involvement, descriptions of these activities are rife with concerns about the ability of food charities to manage escalating food insecurity, arising in the context of widespread poverty, unemployment, and underemployment [2, 3, 7, 30, 31, 33, 34, 36–38],[40, 41].

The prevalence of household food insecurity in Canada has risen significantly since the consistent monitoring of this problem began in 2007 [8]. In 2012, 12.6% of households affected by food insecurity including 4.1% classed as marginally food insecure, 6.0% who were moderately food insecure, and 2.6% who were severely food insecure [8]. In the absence of public policy interventions, the only immediate assistance available to households struggling to meet their food needs is charity, typically provided through food banks. In Canada, food banks are voluntary community organizations that solicit food and financial donations from the public and corporate sectors and distribute food assistance locally, according to whatever principles they have established. Unlike in Europe and the U.S. where public funds are used to augment food bank supplies, in Canada government involvement is primarily facilitative, designed to enable and encourage donations. Patterned after a similar initiative in the U.S., food banks began to proliferate in Canada in the 1980s at a time when public policy responses to poverty and unemployment were being scaled back [23]. The evolution of food banks has, to some extent, been charted through the reports of the national association [42], but membership in this group is voluntary and it does not imply a standardization of operations. From the outset, the organization of charitable food assistance has differed across communities [43], and while there have been studies of the operations of individual food banks [44–46] and specific regional samples of agencies [47–50], there has been no examination of food bank activity at a community level.

The few population- and community-based surveys that have assessed food bank use in Canada indicate that only 20-30% of people experiencing food insecurity seek charitable food assistance [51–56]. A similar discrepancy is evident between household food insecurity prevalence rates and food bank usage statistics: over four million people lived in food insecure households in 2012 [8], but food banks reported assisting 882,188 people [57]. The demographic profile of food bank users also differs from the profile of food insecure households more generally [58]. Food charity is most likely to be accessed by households facing severe food insecurity, but even among this group, fewer than half of households report using food banks [51–53, 55]. Furthermore, some Canadian research suggests that it is common for people who use food banks to still report going hungry, despite receiving food assistance [52, 59, 60], and interviews with vulnerable groups continually highlight their concerns about the nutritional quality, safety, and accessibility of the food assistance provided and the social acceptability of such programs [52, 61–63]. Whether such findings are idiosyncratic, reflecting the limitations of specific food banks, or whether they reflect systemic problems with food banking as a response to chronic problems of household food insecurity in Canada is unclear.

In this paper, we draw on data from a 2010 inventory of charitable food provisioning in five Canadian cities to critically examine the food assistance provided by food banks in these communities. We describe the scope and nature of food bank activities in each city, examine the factors that facilitate and limit food bank operations, and appraise the potential of these initiatives to meet the food needs of those who seek their help.

## Methods

### Selection of cities

The selection of Halifax, Quebec City, Toronto, Edmonton, and Victoria as study sites was designed to facilitate an examination of community-level variation in the organization and operation of food assistance programs. The cities are all provincial capitals, but they vary in size and food insecurity prevalence rates (Table 1). The economic conditions in these cities and the provinces in which they are situated also differ, with Alberta (and Edmonton) enjoying greater prosperity and a lower rate of unemployment than the other study locations [64–66]. Each province has ‘Good Samaritan’ legislation in place that absolves donors of liability for the safety of the food they donate to food banks [67], and some provincial governments have provided grants to support food bank operations [68], but none is directly involved in the coordination or management of food banks.

**Table 1:** **Population, food insecurity prevalence, and scale of food assistance provisioning by city**

	Victoria	Edmonton	Toronto	Quebec City	Halifax	All
Population^a^	358,054	1,176,307	5,741,419	754,358	403,188	
Prevalence of food insecurity^b^	14.0%	13.1%	12.5%	9.0%	19.9%	
Number of agencies providing food assistance	29	68	122	90	31	340
Number of people working (ratio of paid staff to volunteers)	218	479.5	1570.5	580	242.5	3090.5
	(1 : 2.5)	(1 : 4.2)	(1 : 6.4)	(1 : 6.3)	(1 : 4.6)	(1 : 5.3)
Number of people receiving food assistance in one month	12882	16064	90141	11143	7111	137340
Median number served per month by an agency (minimum, maximum)	20	120	266	56	120	120
	(1, 7000)	(2.5, 2450)	(1, 11700)	(2, 1700)	(17, 2100)	(1, 11700)
Agencies supplied by centralized distributor, n (%)	8	47	78	53	27	213
	(27.6%)	(69.1%)	(63.9%)	(58.9%)	(87.1%)	(62.7%)
Proportion of assistance delivered by agencies supplied by a centralized distributor	73.4%	87.3%	77.0%	87.8%	94.4%	79.6%

^a^Statistics Canada, 2010 [69].

^b^Prevalence of marginal, moderate, or severe household food insecurity in 2011-12 for corresponding Census Metropolitan Area [8]. This geographic unit includes, but extends beyond the city.

### Data collection

A comprehensive list of agencies and organizations running charitable food assistance programs in each city was compiled by sourcing, collating, and cross-checking locally available records, including service directories and membership lists for centralized food donation distributors and municipal records of agencies receiving funding for initiatives that might include charitable food assistance. Agencies were eligible to be included in the sample if they provided food free of charge or at nominal cost, in the form of groceries or prepared meals and snacks. Programs in which food access was contingent upon enrolment in a training program or residency (e.g., shelters, group homes, tenant programs) and programs targeted to children were excluded. These exclusion criteria were imposed to ensure that the food assistance programs included provided direct, immediate responses to perceived problems of food insecurity.

A total of 967 agencies were identified, but upon further investigation, 31 were found to have ceased operations and 319 were deemed ineligible. The ineligible agencies included 182 that provided assistance only to specific groups, 80 that were not actually distributing food, 47 that charged more than a nominal fee, and 10 with programs outside the geographic boundaries of our sample. Of the remaining 617 agencies, 517 (84%) consented to participate in a telephone survey. Within cities, response rates ranged from 79% in Toronto to 91% in Victoria and Edmonton. The 100 agencies identified that did not consent to participate include 67 that did not respond to repeated invitations to participate, 18 for which no valid contact information could be obtained, and 15 who were contacted but declined to participate in the survey.

The 45-minute structured interview, conducted with the person identified by the agency as being responsible for the food assistance program(s) in the agency, was designed to elicit information on the scope and nature of the food assistance provided, how these operations were resourced, and the factors that constrained or enabled service delivery. The study was approved by the Human Subjects Research Ethics Board at the University of Toronto.

Although our sample included both agencies serving meals on-site and agencies giving out bags of groceries, the analytic sample for this manuscript includes only agencies providing the latter service. Excluding six agencies that only distributed groceries at Christmas or Passover, the final analytic sample was 340 agencies.

### Data analysis

Descriptive statistics were generated to characterize the scope and nature of food banks in each city. Two sets of multivariate analyses were constructed to elucidate factors that facilitated or limited food bank work.

A series of multivariate regression models were run to identify the resources that predicted the number of people served by an agency. Variables considered included the number of paid staff, number of volunteers, proportion of food distributed that was donated, and binary variables depicting whether or not the agency had funding and whether it engaged in fundraising activities. Non-significant variables were removed sequentially to generate a parsimonious model.

Multivariate analyses were also conducted to examine how agencies’ efforts to meet food needs in their communities related to their ability to consistently deliver their programs. Efforts to meet need were assessed in terms of the number of people helped per month, the frequency with which they provided assistance to individual clients, whether client need had been taken into account in scheduling their services, and whether assistance was provided ‘on demand’. Logistic regression was applied to determine the association between these variables and three indicators of strain in food banks’ abilities to consistently deliver their programs: having to sometimes limit the selection of foods, reduce the amount of food given, and limit people’s access to assistance (i.e., by cutting hours, prioritizing who to serve, or turning people away) because of resource constraints. Models were initially run to generate unadjusted odds ratios for each of these three ‘strain’ variables in relation to the number of people served and binary variables denoting food banks serving more often than once per month, those serving on ‘demand’, and those reporting that their schedules have been developed taking into account the times when food is most needed by their clientele. Multivariate logistic regression models were then run, including all program characteristics, to yield adjusted odds ratios for each ‘strain’ variable in relation to these characteristics. Because there was no significant association between the provision of assistance ‘on demand’ and any indicator of ‘strain’, this variable is omitted from the results presented here.

Because the number of individuals per month receiving assistance was highly skewed, this variable was log-transformed prior to the above-described analyses. All analyses were conducted using SAS statistical software (version 9.2), with SURVEY commands used to account for the clustering of agencies within cities.

## Results

### Scale of operations

The 340 agencies surveyed provided food assistance to almost 140,000 people per month, with the number of people helped in one month ranging from 7,111 in Halifax to 90,141 in Toronto. The scale of operations of individual agencies differed markedly within and between cities, reflecting the extraordinary heterogeneity in these programs. The median number of people served per agency per month was 120, but within agencies this number ranged from 1 to 11,700 (Table 1).

Over half (58.2%) of the agencies surveyed were faith-based organizations (including the Salvation Army and St. Vincent de Paul as well as churches, synagogues, and mosques), and they provided 71.6% of the total food assistance documented. Within cities, the proportion of food assistance that was provided by faith-based organizations ranged from 56.4% in Quebec City to 77.1% in Victoria. There were also differences in the involvement of public sector organizations (e.g., multi-service agencies, health centres, colleges and universities), with such organizations serving 37.7% of people receiving food hampers in Quebec City and 23.7% in Halifax but only 9.8% in Victoria, 13.9% in Toronto, and 15.8% in Edmonton. Every city included some programs that were stand-alone operations, established solely for the purpose of collecting and distributing charitable food assistance, but they provided a relatively small proportion of the total assistance (ranging from 5.9% in Quebec City to 13.5% in Toronto).

**Figure 1:**
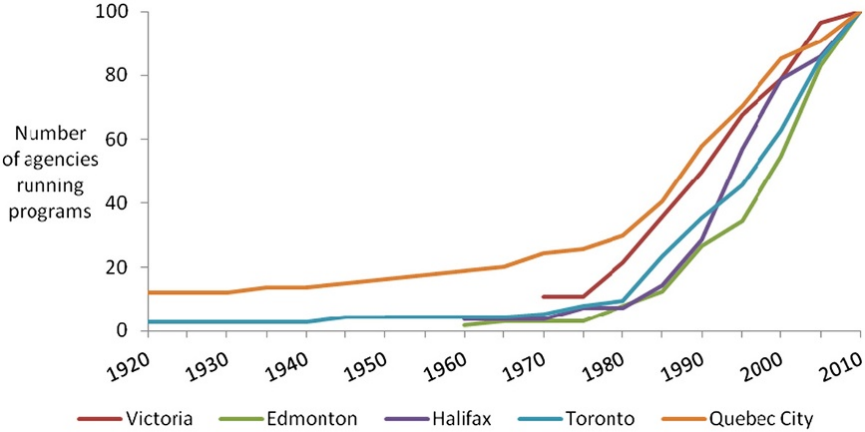
**The initiation of food assistance programs in five cities.**

Sixty-nine percent of agencies surveyed had been providing food assistance for at least 10 years (Figure 1). Quebec City had the longest history of charitable food provisioning, with 25% of agencies surveyed in operation prior to 1970 and five dating back to the 1800s. All five cities exhibited a steady rise in the number of agencies providing food assistance from the mid-1980s onward. Thirty-nine of the 340 agencies surveyed (11.5%), had started providing food assistance in the past five years. Their contribution to the total volume of food assistance provided ranged from 12.6% in Edmonton to less than 1% in Victoria. The presence of new providers suggests that the total scale of charitable food provisioning continues to expand everywhere, but we have no measure of the number of agencies that began but ceased providing food assistance prior to our survey.

### Coordination of food banking

Every city had some organization that coordinated the collection and distribution of donated foods at a local or provincial level and participated in the national food sharing program of Food Banks Canada [70]. These centralized distributors had been in operation for three or four decades, but their structures and operations differed markedly (Table 2), as did their relation to the individual food bank operations within cities (Table 1). In Toronto, the coordinated collection and distribution of donated food was managed by two agencies linked to Food Banks Canada, as well as a third organization that collected perishable food from local retailers and businesses for redistribution. In every city, the majority of food assistance provided in the course of a month was distributed via agencies that were linked to centralized distributors (Table 1).

**Table 2:** **Selected characteristics of major food donation distributors in each city** ^**a**^

	Victoria	Edmonton	Toronto	Quebec City	Halifax
Name	Mustard Seed	Edmonton Food Bank	Daily Bread Food Bank	Moisson Quebec	Feed Nova Scotia
Year began	1975/1985^b^	1981^c^	1983	1987	1984
Origins & Purpose	Christian charity rooted in Baptist church, to fight hunger and restore faith	Edmonton gleaners association, to reconcile waste with hunger	Sisters of St. Joseph (Catholic Order) and others concerned about impact of growing poverty, to fight to end hunger in Toronto communities	Founder André Mignault, to reduce waste and optimize food aid, and provide alternatives to food banks for people living in poverty	Originally Metro Food Bank Society established by faith and corporate communities to provide emergency food relief in Halifax/Dartmouth; now serving the entire province of Nova Scotia.
Services	The largest food bank on Vancouver island assisting 7,000 people/ month; They operate a food hamper delivery program for 10 individuals, a week day drop in with clothing bank and home starter kits, a family centre offering 2 family dinners each month, budgeting, cooking, literacy and parenting support and help to access city services; They also run a church and a faith-based residential recovery program	Their main service is food recovery and distribution of donated food to ~200 agencies; They have an onsite food bank and conduct centralized telephone intake directing clients to one of 40 food depots for pick up, They also provide referrals to food buying co-ops, bread runs, gardens, kitchen, and inexpensive grocers	They operate 2 food banks; collect and distribute food to ~200 member agencies and provide operational support/ guidelines; offer training programs for food bank recipients (food service and catering) and drop-in and shelter volunteers and cooks; they partner in the operation of a community garden; run a referral and information centre; and conduct research to inform practice and advocacy	They collect and distribute food to 140 organizations; provide operational support to agencies wishing to start “food security projects/ alternative practices” including collective kitchens, community gardens, food buying groups; and conduct workshops for food bank workers and recipients.	They collect and distribute donated food to 150 member agencies in communities across Nova Scotia (food banks, meal programs, soup kitchens, school programs and shelters); operate a telephone help line to deal with distress and to inform about nearest food assistance; They also run a culinary training/ employment program; and collect data for advocacy purposes

^**a**^Mustard Seed: http://mustardseed.ca/, Edmonton Food Bank: http://edmontonsfoodbank.com/, Daily Bread Food Bank: http://www.dailybread.ca/, Moisson Quebec: http://www.moissonquebec.com/, Feed Nova Scotia: http://www.feednovascotia.ca/. ^**b**^Mustard Seed began as a small church-based mission in 1975 but expanded and moved to a larger site in 1985. ^**c**^The Edmonton Food Bank was the first official food bank in Canada.

### Food bank resources

While most agencies (82.6%) reported having some funding for their food bank and 48.5% held fundraising events to support their food banks, only 13 agencies purchased all of the food they distributed and they operated on a very small scale (with most assisting fewer than 10 people/month). Almost two-thirds of agencies (62.6%) obtained donated food through centralized distributors, and donations comprised a significantly higher proportion of their food supplies when compared to agencies not linked to centralized distributors (mean 78.1 ± 2.1% among agencies linked to distributors versus 47.3 ± 9.1% among others; F test, p = 0.0240). Independent of whether they obtained food from centralized donation distributors, 46% of the agencies surveyed regularly obtained food donations from local businesses; for the most part, this was food that could not be sold. Two-thirds of agencies who used donated foodstuffs (whether acquired independently from local businesses or via membership in a Food Banks Canada agency) reported sometimes receiving food that was inedible.

Most of the people working in food banks were volunteers (Table 1). Only 52% of food banks reported having any paid worker(s), and volunteers outnumbered paid workers by a ratio of 5.3:1. The presence of paid workers was related not to the size of the food assistance program, but to the nature of the agency in which it was housed. Whereas 90.1% of food banks in multi-service agencies had paid staff working in them, only 38.7% of faith-based service agencies and 28.6% of churches, synagogues, mosques, and other faith centres paid staff to work in their food banks (Rao-Scott Chi-square = 34.3, 5 df, p <0.0001).

### The provision of assistance

Most agencies (78.5%) had regular weekly hours of operation, but most were open only one or two days of the week, typically on Tuesday, Wednesday, and/or Thursday. The availability of food assistance dropped sharply on the weekend in every city, with only 8.5% of agencies operating then. Seventy-three agencies (21.5%) had no fixed hours of operation, and most of these agencies reported providing food assistance ‘on demand’ or ‘as needed’.

One-half of program operators said their programs were intentionally scheduled to provide food when clients were most in need. Across cities, this proportion ranged from 62.2% in Quebec City to 25.8% in Halifax. Agencies that considered clients’ needs when scheduling food bank hours did not have a higher volume of clients, but they were more likely to operate on a Friday, Saturday, or Sunday and to provide food ‘on demand’ (data not shown).

Three-quarters of agencies surveyed allowed people to obtain food assistance at least once per month, with 40% permitting access more often. In general, agencies in Toronto afforded clients more frequent access to food than agencies in other cities, with 39% permitting at least some of their clients to receive assistance on a weekly basis, and no agencies providing food assistance less often than once per month. In contrast, in Quebec City, 30% of agencies surveyed did not permit clients to obtain food assistance monthly, and there were 10 agencies (5 in Quebec City and 5 in Edmonton) that only assisted people once per year.

### Balancing supply and demand

Agencies typically applied eligibility criteria (e.g., income thresholds, residence within a specified ‘catchment’ area, main source of income) when evaluating people’s requests for assistance and placed restrictions on the amount and selection of food given to eligible households as well as the frequency with which any one household could receive assistance. Such routine measures functioned to contain the distribution of food assistance, but when confronted with supply constraints, most agencies invoked additional measures to restrict the assistance distributed. Specifically, 49.4% of agencies sometimes further limited the variety of foods distributed, and 41.2% sometimes reduced the size of hampers because of supply constraints (Table 3). In addition, 27.4% of agencies reported sometimes implementing further measures to restrict access, including prioritizing who they helped (14.7%), turning people away (17.4%), and reducing their hours of operation (8.5%), all because they did not have enough food to maintain their services. In all of these behaviors, there was considerable variation between cities, with the greatest likelihood of restrictions in food access being reported by food bank operators in Toronto, Quebec City, and Halifax (Table 3).

**Table 3:** **Limitations in the delivery of food assistance, by city**

	Victoria	Edmonton	Toronto	Quebec City	Halifax	All
	*n (%)*					
Clients need more food than food bank is able to provide.	17 (58.6)	45 (66.2)	95 (77.9)	61 (67.8)	26 (83.9)	244 (71.8)
Agency would expand food program if more resources were available	17 (58.6)	33 (48.5)	91 (74.6)	50 (55.6)	22 (71.0)	213 (62.7)
Agency sometimes altered the variety of food provided due to lack of food	11 (37.9)	23 (33.8)	76 (62.3)	43 (47.8)	15 (48.4)	168 (49.4)
Agency sometimes cut the size of the hampers provided because of insufficient food	2 (6.9)	16 (23.5)	64 (52.5)	43 (47.8)	15 (48.4)	140 (41.2)
Agency sometimes took additional measures to restrict access^a^	4 (13.8)	10 (14.7)	43 (35.3)	28 (31.1)	8 (25.8)	93 (27.4)

^a^The additional measures assessed included prioritizing who to serve, reducing the hours of service, and turning people away because the agency had insufficient food to meet demands.

Irrespective of whether agencies periodically had to reduce the amounts of food they gave to clients, 72% of program operators said the people they served needed more food than they were able to provide (Table 3). Almost two-thirds of food bank operators said they would expand their programs if they had more resources, but there were notable differences between cities, with 74.6% of food banks in Toronto but only 48.5% in Edmonton expressing a desire to expand. Among program operators who acknowledged that their programs were not doing enough to meet clients’ needs now, 76.5% cited a lack of funding, 67.1% cited lack of staff resources and 62.9% identified the inability to increase their food supplies as preventing them from expanding.

### Facilitating and limiting factors in food bank work

An analysis of the resources associated with the scale of food bank operations revealed two significant predictors of the number of people helped by any one agency in the course of a month: the proportion of food distributed that came from donations (beta 0.0143, SE 0.0024, p 0.0041) and the number of volunteers working in the agency (beta 0.0630, SE 0.0159, p 0.0167). These two variables accounted for 23% of the observed variance in numbers served.

The more food banks did to try to meet food needs in their communities (i.e., by serving more people, serving people more often, and scheduling their services in relation to times of particular need), the more likely they were to report having trouble maintaining their services (Table 4). The odds of a food bank having to sometimes limit the variety of food given, reduce the amount of food given, and restrict people’s access to assistance (i.e., by cutting hours, prioritizing who to serve, or turning people away) rose significantly in relation to the total number of people assisted per month. Food banks serving people more than once per month were more likely to sometimes limit variety and reduce the amount of food given out. Program operators who scheduled their services in relation to perceived needs were also more likely to restrict food access and reduce the amount of food given. When considered simultaneously, the volume of service, the frequency with which clients could receive assistance, and the practice of scheduling services in relation to need were all associated with higher odds of reductions in the amount of food given; the volume of service was associated with higher odds of limiting the variety of food distributed; and the practice of scheduling services in relation to need was associated with higher odds of restricting access to assistance.

**Table 4:** **Odds of an agency having to curtail food bank operations in relation to selected food bank characteristics**

	Reduce variety of food given	Reduce amount of food given	Restrict access to assistance
**Number of people served/month**			
OR (95% CI)^a^	1.34 (1.14, 1.57)	1.27 (1.09, 1.47)	1.16 (1.002, 1.33)
AOR (95% CI)^b^	1.31 (1.10, 1.55)	1.24 (1.04, 1.46)	1.14 (0.98, 1.34)
**Giving food more often than once/month**			
OR (95% CI)	2.14 (1.08, 4.26)	3.33 (1.45, 7.65)	1.74 (0.97, 3.13)
AOR (95% CI)	1.88 (0.89, 4.03)	3.14 (1.31, 7.53)	1.65 (0.89, 3.05)
**Food bank scheduled to accommodate needs**			
OR (95% CI)	1.02 (0.67, 1.57)	1.45 (1.27, 1.65)	1.45 (1.04, 2.02)
AOR (95% CI)	1.16 (0.74, 1.82)	1.71 (1.40, 2.10)	1.56 (1.06, 2.29)

^a^Odds ratios and 95% confidence intervals have been derived from logistic regression analyses.

^b^Adjusted odds ratios and 95% confidence intervals have been derived from a single multivariate logistic regression including a continuous variable for the log-transformed number of people served and binary variables to denote food banks serving more often than once per month and those reporting that their schedules have been developed taking into account the times when food is most needed by their clientele.

## Discussion

In every city studied, we found extensive, well-established food bank activity, although the delivery of services differed considerably between agencies. The provision of food assistance was fuelled by food donations and volunteer labour. The majority of agencies surveyed indicated that clients needed more food than they provided. Yet the harder food bank operators tried to meet clients’ food needs, the more likely they were to report having to limit food selection, reduce the amount of food given out, and deny people assistance. Put another way, food bank work only appeared to achieve equilibrium between supply and demand when demand was contained. This ‘containment’ was evident in our data through restrictions on the frequency with which clients could access assistance and limits on the scheduling of services. When access to assistance was less restricted, the odds of food banks running out of food and invoking measures to ration remaining supplies and restrict access rose significantly.

Prior Canadian research has described how the donor-driven nature of food banks shapes both the quantity and nutritional quality of food available for distribution, setting the stage for restrictions on the frequency, amount, and selection of food assistance available to clients [48, 49]. Evaluations of the food provided by food banks have consistently documented limited quantities and poor nutritional quality [44, 46], and research with food insecure households has repeatedly highlighted their perception that food banks are unresponsive to their needs [52, 61–63, 71]. In contrast to this research, we set out to study food bank activity at a community level and through that lens to explore the dynamic relationship between the provision of charitable food assistance and requests for help. Our finding that food banks’ policies and practices to manage demand were inextricably linked to their experiences of strain suggests that the tension between supply and demand charted through smaller regional studies is in fact generalizable to the system overall.

Our study is not without limitations. The inventories of local charitable food provisioning activity that we constructed may have been incomplete, and 16% of identified agencies did not participate in this study. Additionally, we excluded 182 agencies that only provided food assistance to specific groups (e.g., participants in particular programs, school children), and this is no doubt not an exhaustive list of such programs. By focusing this study on food bank programs providing assistance to the general public, we have characterized only one part of charitable food provisioning in the cities studied. Moreover, we have no measure of the quantity of food distributed in the agencies surveyed or the length of time over which they have assisted individual clients, and no way of knowing how well any of the agencies surveyed were meeting their clients’ food needs. Our appraisal of food bank activity relies on self-reports of the numbers of people served and the resources deployed to provide these services. Not all agencies maintain detailed records, and thus our data must be subject to estimation errors. Our study is also limited insofar as we restricted our inquiry to the provision of food assistance. Many food banks’ activities extend beyond food assistance [42, 50], and the agencies surveyed may have provided additional services and supports that directly or indirectly impacted their clients’ food security (e.g., employment assistance, referrals to other service providers, food skills). While food assistance is the most direct response to problems of food insecurity, in interpreting our findings, it is important to recognize that food may not have been the only assistance given to the people who used these services.

Changes in food bank usage are widely interpreted to indicate changing levels of food insecurity or food poverty, particularly in European countries and the United Kingdom where food insecurity is not routinely monitored [7, 35, 39–41, 72], but also in Canada where the tracking of food bank data predates food insecurity monitoring and Food Banks Canada continues to release an annual report on food bank utilization entitled ‘HungerCount’ [42]. The results of our study lend support to recently published critiques of this use of food bank statistics [58, 72] by providing empirical evidence that the number of people served by food banks is in part a function of how much usage food banks will permit. Simply totaling up the numbers served by different agencies gives the illusion that they are providing equivalent services, and more importantly, that all agencies are equally responsive to changing needs within the communities they serve. Our findings suggest that in the five cities studied demand may have been artificially contained by the restrictive distribution practices in some agencies, and that even in agencies whose operations were designed to more fully meet needs, the provision of assistance was sometimes curtailed because demand exceeded supply. Moreover, two-thirds of agencies surveyed indicated that they would like to expand their services, but resource constraints precluded this. These observations imply that the number of people served by food banks is not a sensitive measure of food needs in a community. While our data derive from five Canadian cities, the insights drawn from our research have implications for the interpretation of food bank utilization statistics in other settings where the distribution of food assistance is supply-driven and constrained by resource limitations and arbitrary restrictions on access.

Since their inception, food banks have been the backbone of community responses to problems of household food insecurity in Canada, despite the early appraisal that they were meant to be a temporary solution to acute crisis [23, 43, 73, 74]. The shift from temporary response to an enduring feature of the urban environment in the context of persistent and growing food insecurity is evident in the long histories of charitable food provisioning among most of the agencies we surveyed. This shift invites long hard questions about the effectiveness of this response. Our study findings indicate that food banks remain dependent on donations and volunteer labour, with available resources quickly exhausted in the face of agencies’ efforts to more fully meet clients’ needs. This analysis contradicts the prevailing notion that food banks are an effective short-term response to problems of acute food need in our communities, and that they are somehow capable of managing at least the symptoms of severe food insecurity (‘hunger’) locally [60]. It could be argued that introducing measures to increase the supply of food to food banks, such as the introduction of tax credits for corporate donors that has been proposed by Food Banks Canada [75] or the allocation of public funds to purchase food to augment food supplies (e.g., similar to The Emergency Food Assistance Program in the U.S. [31] or the funds disbursed by the European Commission to provide material assistance to the ‘Most Deprived’ [76]), would enable food banks to distribute more food. However, such actions would reinforce the idea of food charity as an appropriate response to food insecurity in Canada without seriously engaging in the question of its effectiveness. The charitable model of food distribution that defines food bank activity makes it difficult, if not impossible, for agencies to gauge the impact of their work in relation to need [49], and the population-representative surveys that yield measures of household food insecurity in Canada do not assess food bank use. Yet, our findings raise serious questions about the capacity of food banks to respond to the needs of the people who seek their help. Any actions to expand food bank activity need to be accompanied by measures to evaluate the impact of these programs on the problems of food insecurity that they are intended to address.

## Conclusions

Direct extrapolations from our analysis to food bank activities in other countries are limited by the context-dependent nature of this activity, but the question of how well food banks are responding to local problems of food insecurity is of paramount importance to critical analyses of these systems in all jurisdictions. Food insecurity is expected to grow with the continued global economic instability, ecological and resource constraints on continued economic growth, rising energy prices (e.g. impact of fuel costs on food distribution systems), government policy responses (fiscal austerity), and the anticipated impact of climate change on food production (and prices) in the coming decade [77]. Thus it is critical that governments implement effective responses. As the prevalence of food insecurity continues to rise in Canada, we contend that primacy must be given to policy interventions that tackle the material deprivation that underpins this condition, with proactive government involvement to ensure accountability toward this goal.
